# Near-Infrared Fluorescence Imaging Directly Visualizes Lymphatic Drainage Pathways and Connections between Superficial and Deep Lymphatic Systems in the Mouse Hindlimb

**DOI:** 10.1038/s41598-018-25383-y

**Published:** 2018-05-04

**Authors:** Yukari Nakajima, Kimi Asano, Kanae Mukai, Tamae Urai, Mayumi Okuwa, Junko Sugama, Toshio Nakatani

**Affiliations:** 1 Department of Clinical Nursing, Graduate Course of Nursing Science, Division of Health Sciences, Graduate School of Medical Sciences, Kanazawa University, Kanazawa, Japan; 2 Research Fellow of Japan Society for the Promotion of Science, Tokyo, Japan; 3 School of Nursing, Kanazawa Medical University, Uchinada, Japan; 4 Faculty of Health Sciences, Institute of Medical, Pharmaceutical and Health Sciences, Kanazawa University, Kanazawa, Japan; 5 Advanced Health Care Science Research Unit, Innovative Integrated Bio-Research Core, Institute for Frontier Science Initiative, Kanazawa University, Kanazawa, Japan

## Abstract

Since lymphedema rarely develops in the mouse hindlimb, the underlying mechanisms remain unclear. We herein investigated the resolution of chronic hindlimb lymphedema in mice using a Near-Infrared Fluorescence (NIRF) imaging system. Nineteen 7–28-week-old BALB/c male and female mice were injected with two dyes for lymphography and dissection. Lymphadenectomy was performed on six male mice to completely obstruct lymph flow in the hindlimb. Edematous changes in both hindlimbs were compared until 60 days after surgery. The NIRF imaging system detected three lymphatic collecting systems in the mouse hindlimb: superficial lateral, superficial medial, and deep medial. It also showed connections between the superficial and deep lymphatic systems in the inguinal region. Lymphadenectomy of the iliac, inguinal, and popliteal lymph nodes caused edematous changes. However, lymph flow in these operated areas restarted within 60 days and the severity of lymphedema appeared to be low. NIRF imaging showed that the deep medial system and a connection between the superficial and deep lymphatic systems in the inguinal region drain lymph from the hindlimb. This is the one reasons why lymphedema does not develop in the mouse hindlimb. The stable obstruction of lymph flow in these three systems is desired to develop chronic lymphedema.

## Introduction

The lymphatic system functions as a drainage system of fluid as well as an immune system by controlling immune responses. It consists of lymphatics and lymph nodes (LNs), and plays important roles in the pathogenesis of several diseases, such as lymphedema, cancer metastasis, and various inflammatory conditions. Secondary lymphedema results from direct injury to the lymphatic system after surgery or radiotherapy, and, as a consequence, lymph fluid accumulates in subcutaneous tissue^1^. Lymphatic stasis in subcutaneous tissue causes inflammation, resulting in CD4+ cell inflammation and the infiltration of mature T-helper cells, and patients with severe lymphedema ultimately develop fibrosis^2^. However, the underlying mechanisms have not yet been elucidated in detail^3^. Treatments for lymphedema, including lymphatico-venous anastomosis (LVA)^4^ and vascular endothelial growth factor-C/D (VEGF-C/D) therapy^5^, have been conducted in laboratories and clinical settings. LVA involves microsurgery that anastomoses lymphatic vessels and veins in subcutaneous tissue. However, the long-term patency of LVA is limited, and appears to be due to contact between lymphatic endothelial cells (LECs) and platelets. Podoplanin, which is expressed in LECs, is a ligand for the C-type lectin-like receptor (CLEC-2) of platelets. CLEC-2 releases bone morphogenetic protein-9 (BMP-9) when activated^6^. A previous study reported that platelets, mainly BMP-9, regulate blood/lymphatic vessel separation by inhibiting the proliferation, migration, and tube formation of LECs^7^. This finding proposes a reason for why the long-term patency of LVA is limited. Furthermore, VEGF-C therapy has been shown to promote the lymphatic metastasis of tumor cells^3^. Radical treatments for lymphedema and their evaluation have not yet been established. Therefore, the establishment of a lymphedema model is desired for the development of effective treatments for patients.

Animal models of lymphedema have been previously reported. Radiation therapy is often used to generate lymphedema models. However, we consider this therapy to be unsuitable for the generation of a lymphedema model because it is associated with immobility and high mortality^8,9^. A tail lymphedema model may be used as a limb lymphedema model to elucidate the underlying mechanisms of or evaluate therapeutic approaches for lymphedema because its anatomy and the surgical techniques involved are simple and reproducible^10^. The superficial and deep lymphatic systems of the tail are disrupted, a 2–3 mm portion of the skin is then circumferentially excised, and the deep lymphatic system is microsurgically ligated. After surgery, tail lymphedema persists for three weeks without necrosis or any side effects^11^. However, Frueh *et al*.^10^ indicated that fluctuations in the duration and robustness of this lymphedema depend on the surgical technique used. Furthermore, patients with secondary lymphedema undergo lymphadenectomy. After lymphadenectomy, the risk of infection (lymphangitis and cellulitis) generally increases, while immune responses become weaker^12^. However, only lymphatic vessels are excised in a tail lymphedema model. Consequently, limb lymphedema appears to be more appropriate than tail lymphedema for elucidating the underlying mechanisms and evaluating therapies. Previous studies performed lymphadenectomy on inguinal and/or popliteal LNs in order to achieve stable lymphedema in the hindlimb^9,13^. However, hindlimb edema only occurred transiently after the excision of inguinal and/or popliteal LNs. It currently remains unclear why hindlimb lymphedema does not occur, and this may be attributed to the lack of a clear understanding of the lymphatic system in animals. Hence, the surgical anatomy of the lymphatic system needs to be investigated in more detail.

Near-infrared fluorescence (NIRF) imaging has recently been introduced to visualize lymphatic and blood vessels^3,14^. The mapping of lymphatics before lymphatico-venous anastomosis, sentinel LN mapping^15^, and the classification of lymph flow patterns^16^ are performed using the NIRF imaging system in clinical settings to visualize lymphatics. These approaches provide real-time guidance on the location of the lymphatic system. In addition, NIRF easily detects lymphatics, which are visible to the naked eye, without dissection or special techniques. Therefore, NIRF imaging is beneficial for the visualization of lymphatic drainage pathways and localization of the lymphatic system.

The purpose of the present study was to obtain detailed information on the lymphatic system in the mouse hindlimb using the NIRF imaging system. A clearer understanding of the lymphatic system is important for achieving stable lymphedema.

## Results

### Detection of Lymphatic Vessels Using the NIRF Imaging System

**Figure 1:**
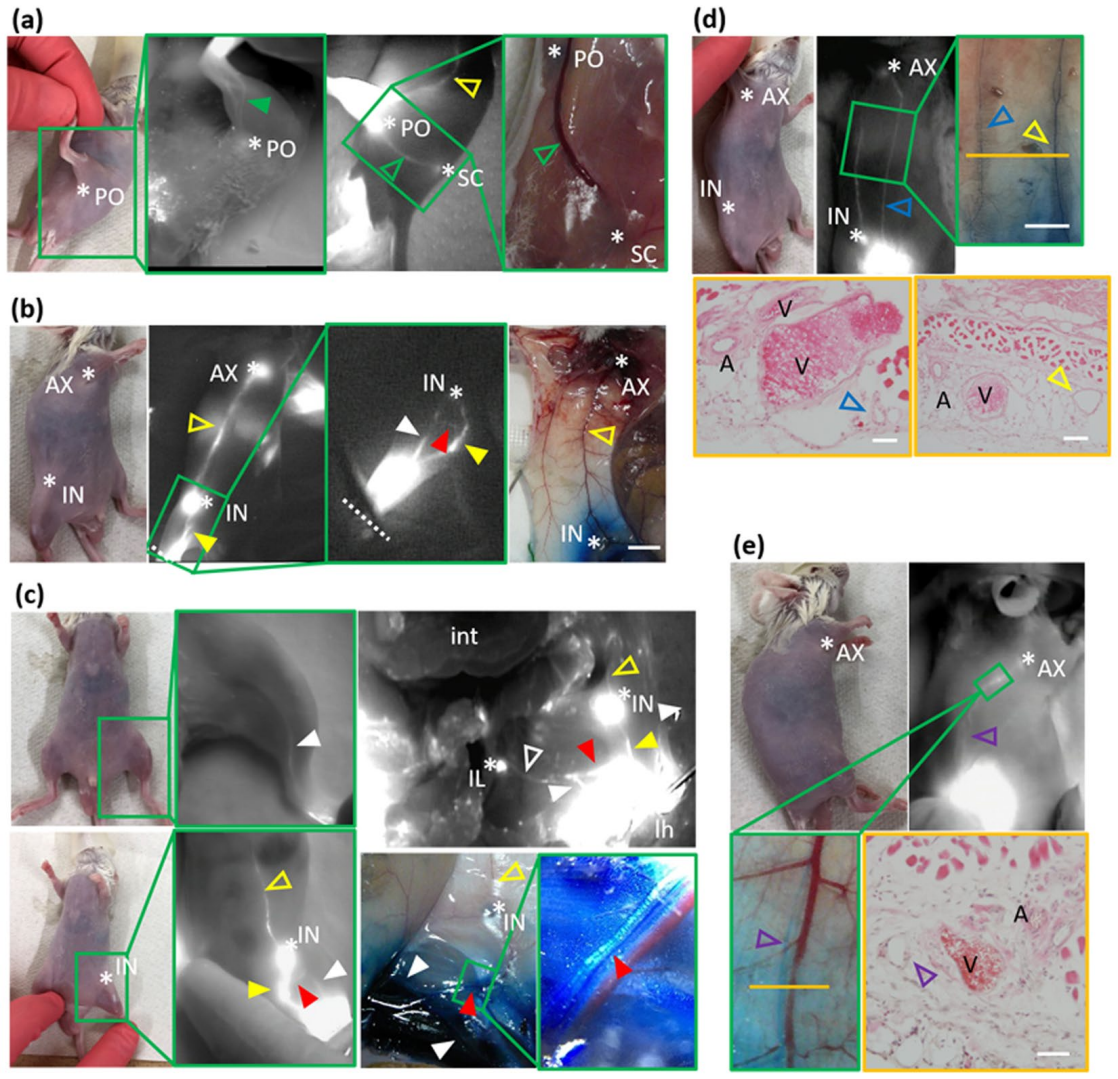
Lymphatic systems detected by visual images, NIRF, and photomicrographs. Visual images of mice on (**a**) to (**e**) revealed each location of right NIRF images. Green large boxes showed the high magnification of each site in green small boxes. (**a**) The superficial lateral system passed the calf muscle (closed green arrowhead) through the popliteal lymph node (LN) and onto the sciatic LN (open green arrowhead), left hindlimb, injection site; green arrow in Fig. 2. (**b**) The superficial medial system passed into the thigh and through the inguinal LN (closed yellow arrowheads), and then ran into the axillary LN (open yellow arrowheads), right hindlimb. Injection site; yellow arrow in Fig. 2, dashed white line; the level of the ankle, Scale bar; 1 cm. (**c**) Upper images; the deep medial system passed into the thigh and entered the abdominal cavity (closed white arrowheads), then ran into the iliac LN (open white arrowheads), left hindlimb. The right image was obtained after dissection of the abdominal cavity. Injection site; gray arrow in Fig. 2. Bottom images; another lymphatic that contributes to the deep medial system (closed red arrowheads) was detected between the inguinal and iliac LNs by the injection of dyes at the site indicated by the yellow arrow in Fig. 2. (**d**) A newly detected lymphatic from the lower abdomen to the axillary LN (open blue arrowhead), left lateral position. Injection site; blue arrow on the left figure in Fig. 2. Hematoxylin and eosin (H&E)-stained images; cross-sectional images at the level of the orange line in the upper right image. Scale bars; upper right 1 cm, bottom left 50 µm, bottom right 100 µm. (**e**) Lymphatics arising from the lower back and running to the superficial axillary LN (open purple arrowheads), left lateral position. A H&E image was obtained at the level of the orange line in the left bottom image. Injection site; purple arrow on the right fig. in Fig. 2. Scale bar; 50 µm. *AX; axillary LN, *IN; inguinal LN, *IL; iliac LN, *PO; popliteal LN, *SC; sciatic LN, A; artery, V; vein, int; intestine, lh; left hindlimb.

We performed lymphography, dissection, and histological observations in order to identify the location at which lymph fluid in the hindlimb is drained. Figure 1 shows the lymphatic collecting system of the hindlimb and above the level of the hindlimb from lymphographic, macroscopic, and microscopic images of intact mice. Three main lymphatic collecting systems were identified in the hindlimbs of mice: (1) superficial lateral; (2) superficial medial; (3) deep medial. We named these systems based on a previous study that examined the normal anatomy of lymphatics in the dog hindlimb^17^. There is no variation in these lymphatic systems of the hindlimb between different animals of the BALB/c strain. All lymphatics detected in the present study were ipsilateral and without sex or age differences.

**Figure 2:**
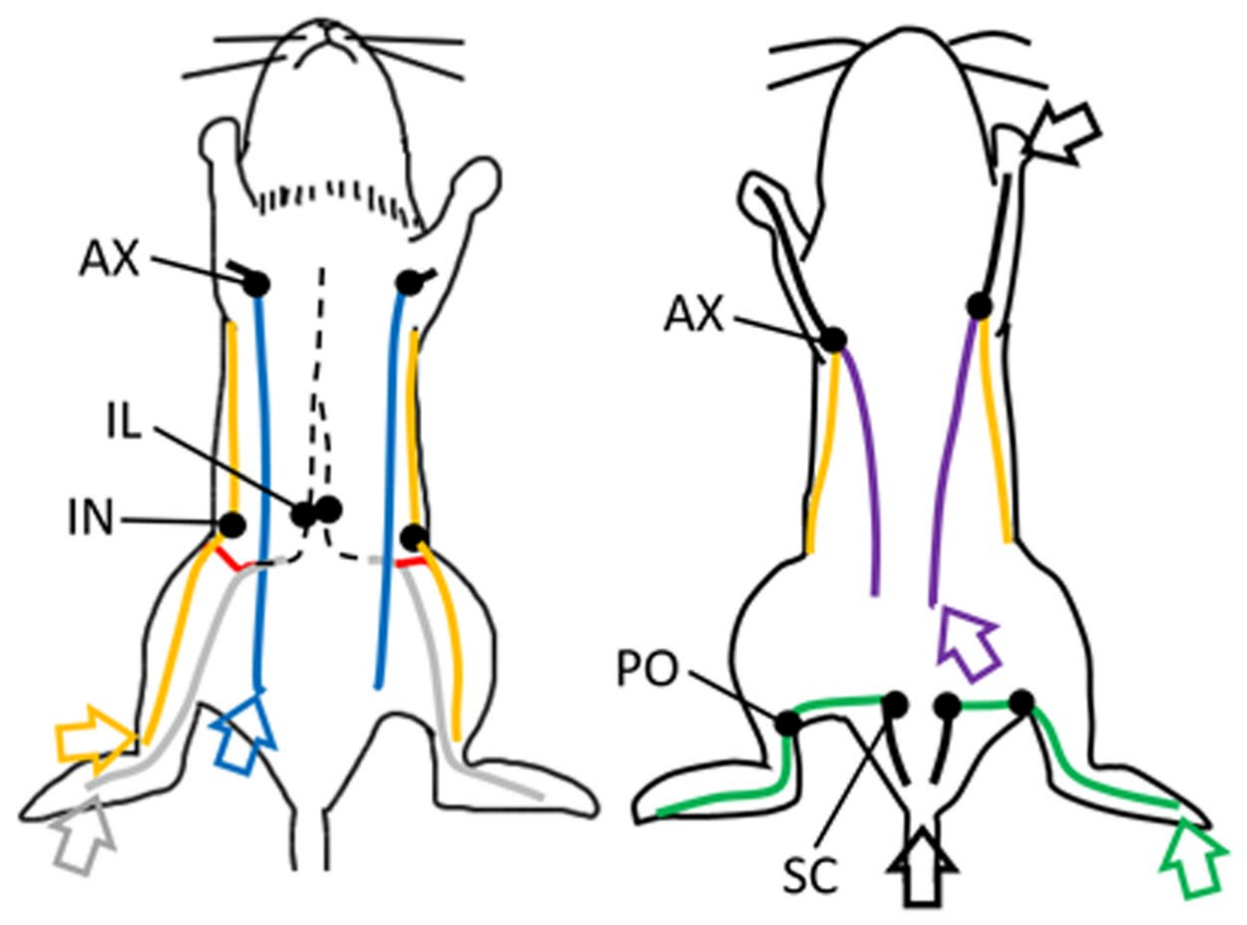
Schematic drawing of the lymphatic system related to the drainage of lymph fluid in the hindlimb of mice. This figure was based on results obtained from lymphography and the dissection of mice. Black circles; lymph nodes (LNs), dashed black lines; lymphatics in the intra-abdomen or intrathoracic area, black solid lines; lymphatics in subcutaneous tissue or muscle on the upper limb or buttock, green solid lines; the superficial lateral system corresponding to open and closed green arrowheads in Fig. 1a, yellow solid lines; the superficial medial system corresponding to open and closed yellow arrowheads in Fig. 1b, gray solid lines; the deep medial system corresponding to open and closed white arrowheads in Fig. 1c, red solid line; lymphatics that started at the level of the inguinal region and ran into the iliac LN corresponding to closed red arrowheads in Fig. 1c, blue solid lines; lymphatics that started at the lower abdomen and ran into the axillary LN corresponding to open blue arrowheads in Fig. 1d, purple solid lines; lymphatics that started from the lower back and ran into the axillary LN corresponding to open purple arrowheads in Fig. 1e. Open green arrow; the injection site of dyes that visualized the superficial lateral system, open yellow arrow; the injection site of the dyes that visualized the superficial medial system and lymphatics revealed by red solid lines, open gray arrow; the injection site of the dyes that visualized the deep medial system. The open gray arrow is the same site as the open green arrow. Left figure; supine position, right figure; prone position. IN; inguinal LN, AX; axillary LN, IL; iliac LN, PO; popliteal LN, SC; sciatic LN.

Figure 1a shows the superficial lateral system of the left hindlimb of a mouse. It started as a network on the foot pad and then crossed to the lateral side of the hindlimb at the level of the lower one third or middle of the calf (closed green arrowhead). This system passed through the popliteal LN to the sciatic LN along with blood vessels (open green arrowhead). It was detected by an intradermal injection of ICG into the foot pad. The injection site was indicated as a green arrow in Fig. 2.

Figure 1b shows the superficial medial system of the right hindlimb of a mouse. It appeared to start above the level of the ankle (closed yellow arrowhead). It was not possible to observe this system under the level of the ankle because it was detected by a subcutaneous injection of ICG into the ankle. This system passed into the thigh and through the inguinal LN (closed yellow arrowheads), and then ran into the axillary LN along with blood vessels in the abdomen (open yellow arrowheads). This system was detected by a subcutaneous injection of ICG into the ankle. The injection site was indicated as a yellow arrow in Fig. 2.

Figure 1c showed the deep medial system of the left side in a mouse. It started at the interior part of the hindlimb, ran with the femoral artery into the thigh, entered the abdominal cavity (closed white arrowheads), and then ran into the iliac LN (open white arrowheads). This system was detected by an intradermal injection of dyes into the foot pad. The injection site was indicated as a gray arrow in Fig. 2. Lymph fluid drained into the abdominal cavity through this system (Supplemental video 1). Another lymphatic that contributed to the deep medial system was detected by the NIRF imaging system during the experiment. After a subcutaneous injection of dyes into the ankle, this lymphatic was observed by stretching the skin to the sagittal line (closed red arrows in Fig. 1c, Supplemental video 2). The injection site was indicated as a yellow arrow in Fig. 2. After dissection, this lymphatic appeared to start at the level of the inguinal region and ran into the iliac LN with blood vessels and nerves (closed red arrows in Fig. 1c). Furthermore, this lymphatic was surrounded by subcutaneous tissue and easily destroyed. Lymph fluid from the superficial medial system drained into the deep medial system through this lymphatic (Supplemental video 2). This lymphatic appeared to start from the inguinal LN or branched from the superficial medial system. However, difficulties were associated with precisely identifying its origin in the present study.

The NIRF imaging system detected two lymphatics that have not been reported in previous studies, as shown in Fig. 1d (open blue arrowheads) and 1e (open purple arrowheads). The first lymphatic, which started at the lower abdomen and ran into the axillary LN (open blue arrowheads), was detected by an ICG injection into the lower abdomen (blue arrow on the left figure in Fig. 2). Mice were found to have two large lymphatics in the abdomen. These lymphatics were also observed histologically (bottom images in Fig. 1d). Second lymphatics, which started from the lower back and ran into the axillary LN (open purple arrowheads in Fig. 1e), were detected by an ICG injection into the lower back (purple arrow on the right figure in Fig. 2). This lymphatic was also observed histologically (right bottom image in Fig. 1e). Figure 2 shows a schematic drawing of the lymphatic system detected by NIRF in the present study. Based on this lymphatic system, operated areas were selected to obstruct lymph fluid in the hindlimb.

### Lymphedema Formation and the Impact of Lymphadenectomy in the Hindlimb

**Figure 3:**
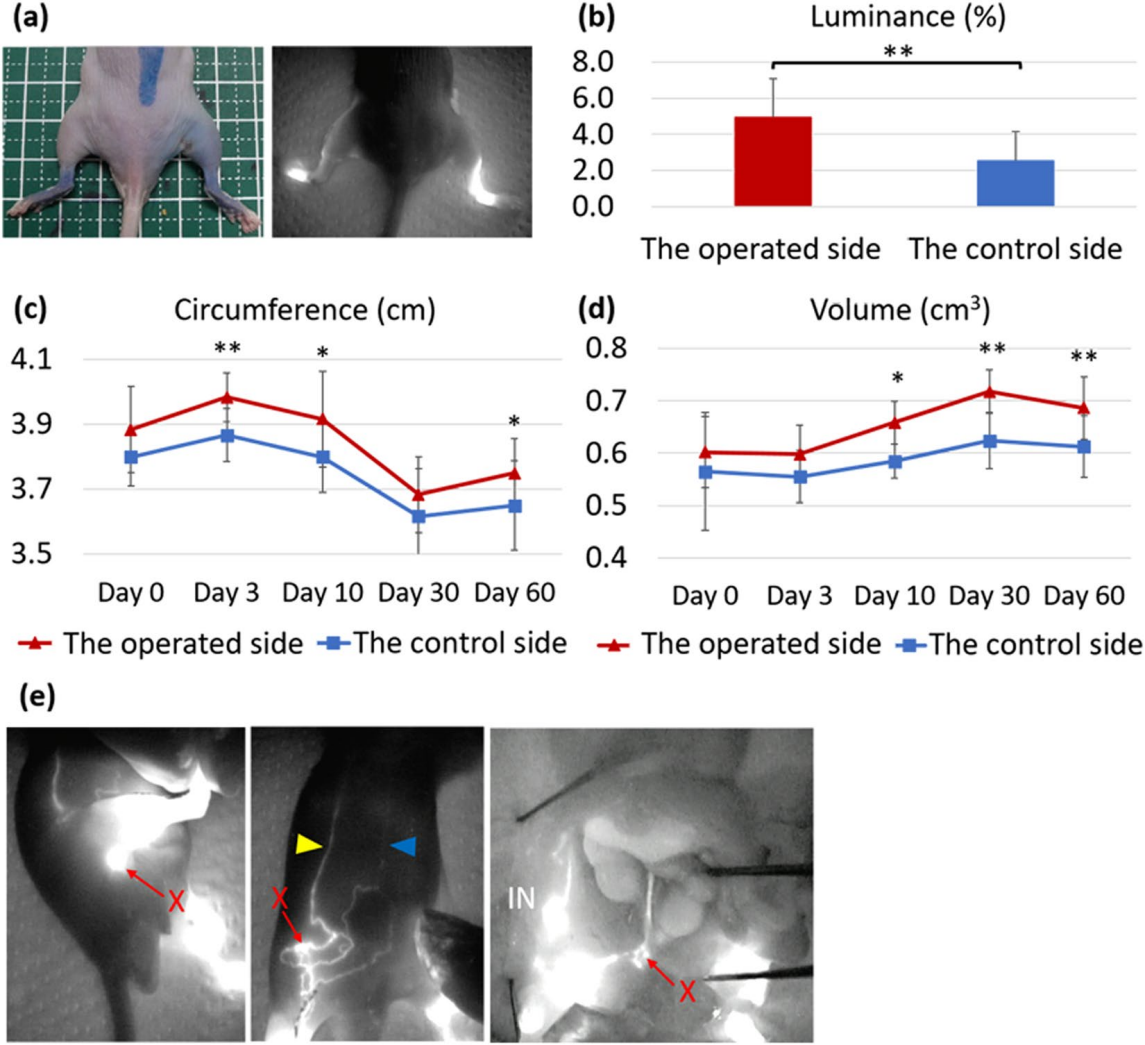
Macroscopic observations after lymphadenectomy. (**a**) Representative images of mice subjected to lymphadenectomy, right hindlimb; the operated side, left hindlimb; the control side. (**b**) The ratio of luminance on day 60. A 2-μl injection of indocyanine green (ICG) was performed on both hindlimbs on day 58. (**c**) Changes in hindlimb circumference until day 60. (**d**) Changes in hindlimb volume until day 60. (**e**) Representative images of lymphography obtained from operated areas on day 60 after lymphadenectomy. Left image; operated areas from which popliteal lymph nodes (LNs) were excised after a 2- to 10-μl injection of ICG at both food pads, center image; operated areas from which inguinal LNs were excised after a 2- to 10-μl injection of ICG at both ankles, right image; operated area from which iliac LNs were excised after a 2- to 10-μl injection of ICG at both food pads. In some mice, lymph flow connected two lymphatics in the abdomen (yellow and blue arrowheads) after passing the hindlimb. Red crosses; operated area, IN; inguinal LN. N = 6 in b, c, d and e. *P < 0.05 and **P < 0.01 versus the control (paired *t*-test).

In order to obstruct lymph fluid in the hindlimb, the inguinal, popliteal, and iliac LNs were excised by surgery because these LNs are located on the three lymphatic systems in the hindlimb. The operated side showed edematous changes 60 days after lymphadenectomy. Macroscopically, remaining Evans blue dye, which was injected on the operated day, was clearly observed in the operated side (Fig. 3a), although the control side received the same dose of dye on the same day. After the injection of ICG into the ankle on day 58, the rate of luminance was calculated on day 60. It was higher on the operated side than on the control side (Fig. 3b). Furthermore, the hindlimb circumference and volume were higher on the operated side than on the control side on day 60 (Fig. 3c,d).

Lymphography was performed on day 60 on both hindlimbs after the ICG injection in order to clarify whether lymph flow in the operated areas was obstructed by surgery. The control side in each animal showed normal lymph flow. However, in the operated area from which the popliteal LN was excised, lymph flow was obstructed in approximately 85% of mice (left image in Fig. 3e). Lymph fluid in the superficial lateral system drained into the sciatic LN in one mouse after excision of the popliteal LN. In operated areas from which the inguinal LN was excised, lymph flow was obstructed in approximately 16% of mice. Tortuous lymph flow was detected around the operated area. Mice showed well-developed detours toward lymphatics of the abdomen after surgery. The detours around this operated area connected two lymphatics in the abdomen in two mice (central image in Fig. 3e) and one lymphatic in the abdomen in three mice. Lymph fluid through the superficial medial system passed these detours and was then drained into the deep or superficial axillary LN. In operated areas from which the iliac LN was excised, lymph flow passed into the deep medial system, accumulated in the operated area, and then entered into the thoracic duct (right image in Fig. 3e). Detours were not observed around this operated area. Lymph flow in this area restarted in all mice.

**Figure 4:**
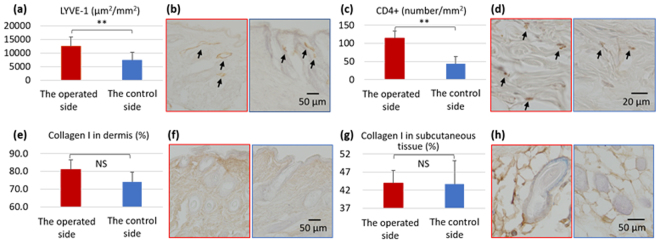
Histological evaluation of the hindlimb after lymphadenectomy on day 60. (**a**) The area of capillary lymphatics in 1 mm^2^ of the dermis. (**b**) Representative photomicrographs of LYVE-1 immunohistochemical staining in the operated side (left) and control side (right). (**c**) The number of CD4+ cells in the dermis and subcutaneous tissue in 1 mm^2^. (**d**) Representative photomicrographs of CD4 immunohistochemical staining in the operated side (left) and control side (right). (**e**) The ratio of collagen I in the dermis. (**f**) Representative photomicrographs of collagen I immunohistochemical staining in the operated side (left) and control side (right). (**g**) The ratio of collagen I in subcutaneous tissue. (**h**) Representative photomicrographs of collagen I immunohistochemical staining in the operated side (left) and control side (right). Black arrows; lymphatics (**b**), CD4+ cells (**d**). N = 5 in a, c, e, and g. **P < 0.01 versus the control (paired *t*-test).

Histological findings on the operated side were compared to those on the control side in order to identify the impact of lymphadenectomy. The area of capillary lymphatics detected by the anti-LYVE-1 antibody was greater on the operated side than on the control side (Fig. 4a,b). The number of CD4+ cells was two-fold higher on the operated side than on the control side. These results revealed that local inflammation occurred after lymphadenectomy (Fig. 4c,d). The ratios of collagen I in the dermis and subcutaneous tissue were calculated in order to evaluate the degree of fibrosis. No significant differences were observed between the two sides (Fig. 4e to h).

## Discussion

In the present study, NIRF detected three lymphatic collecting systems in the mouse hindlimb. The superficial lateral and superficial medial systems have already been reported^18–20^. These lymphatic systems were also observed in the rat^13^. Previous studies excised the popliteal or/and inguinal LNs in an attempt to obstruct these systems and develop hindlimb lymphedema. However, the resolution of lymphedema in the hindlimb gradually occurred after several weeks by surgery only^9,13^. The present study showed that the deep medial system and lymphatics between the inguinal and iliac LNs drain lymph fluid in the hindlimb to the iliac LN, similar to the superficial lateral or superficial medial system. The present results strongly suggest that this is the one of the reasons why hindlimb lymphedema does not develop following surgery. Hence, we need to obstruct not only the popliteal and inguinal LNs, but also the iliac LN in order to develop hindlimb lymphedema. In the present study, lymphatics between the inguinal and iliac LNs were detected in all mice. Lymphography of these lymphatics showed that these lymphatics drain lymph fluid from the superficial to deep lymphatic systems, and, thus, are a connection between these systems. This connection has not been reported in previous studies that examined animals. In humans, no connections between the superficial and deep lymphatic vessels were observed in the inguinal region^21–23^. Connections between the superficial and deep lymphatic systems have only been reported above the elbow along the basilic vein^24^ and popliteal region in cadavers^23^. The connections between the superficial and deep lymphatic systems differ between species. Therefore, this result is very valuable for understanding not only the lymphatic system, but also differences in lymphatic systems between species.

We performed lymphadenectomy in order to interrupt lymph drainage in the hindlimb. The operated side showed an increased hindlimb circumference, volume and stasis of lymph fluid, inflammation, and lymphatic area. These edematous changes have been reported previously^2,25,26^. The circumference of the hindlimb and the volume and stasis of lymph fluid were measured in order to evaluate the extent of lymph edema^20,27,28^. These parameters all increased in edematous tissue. In the histology of edematous tissue, lymphatic stasis causes CD4+ cell accumulation in subcutaneous tissue and leads to inflammation^2,29^. Furthermore, the average cross-sectional areas of lymphatics significantly increase as a result of lymphedema responses^26^. Therefore, lymphadenectomy of the popliteal, inguinal, and iliac LNs appears to produce stable lymphedema in the hindlimb. However, in clinical settings, patients with severe lymphedema ultimately develop fibrosis^30^. In the present study, lymphadenectomy of the popliteal, inguinal, and iliac LNs did not cause fibrosis. We attributed this result to lymph flow in areas in which LNs were excised restarting within 60 days of surgery. Lymph fluid was not completely obstructed in the present study. Hadrian *et al*.^31^ reported that rodent models showed high endogenous lymphatic regeneration; therefore, stable lymphedema rarely developed. Hindlimb lymphedema in mice may not be produced by surgery only. We consider the stable obstruction of lymph fluid in the three lymphatic collecting systems achieved in the present study using other approaches to result in the development of chronic lymphedema in mice. Further studies are needed in order to induce the stable obstruction of these lymphatic systems.

Advances have recently been achieved in the development of measuring devices. However, current knowledge on the anatomy of the lymphatic system in mice is largely based on studies conducted before 2000. In the present study, we used NIRF to identify lymphatic systems, and it successfully detected novel lymphatics. A better understanding of the anatomy of the lymphatic system using recently developed devices will lead to more effective treatments, care, and the prevention of iatrogenic lymphedema by surgery in patients with lymphedema.

### Study limitations

Lymph draining through interstitial flow was not evaluated in the present study. Although lymph in the hindlimb was partly drained by interstitial flow, we were unable to establish how much lymph flowed interstitially and what its impact was. This study was conducted using BALB/cCrSlc mice. There is very limited information on the anatomy of lymphatic systems in the hindlimb in other strains of animals. Therefore, the lymphatic system needs to be investigated in lymphedema models of other strains of animals. Since NIRF does not have the ability to detect the deep lymphatic system in the abdominal cavity, when the deep lymphatic system is being examined, dissection is needed to confirm lymph flow.

## Conclusions

NIRF detected three lymphatic collecting systems in the mouse hindlimb: the superficial lateral, superficial medial, and deep medial collecting lymphatic systems. Lymphatics between the inguinal and iliac LNs represent a connection between the superficial and deep medial lymphatic systems in mice. Lymphadenectomy of the popliteal, inguinal, and iliac LNs resulted in stable lymphedema in the hindlimb; however, its severity appeared to be low. Further studies are needed in order to completely obstruct lymphatic systems for the development of a severe lymphedema model.

## Methods

All procedures involving animals were reviewed and approved by the Committee on Animal Experimentation of Kanazawa University (AP-163811). The methods were performed in accordance with the approved guidelines.

### Mice

Twenty-five BALB/cCrSlc male and female mice (Sankyo Lab Service Corporation, Inc., Toyama, Japan) weighing 19.7–30.7 g were used at 7 to 28 weeks of age. They were caged individually in an air-conditioned room at a temperature of 25.0 ± 2.0 °C, and the lights were kept on between 08:15 and 20:15 hours. Water and pelleted food were given freely.

Nineteen male and female mice aged 7 to 28 weeks were subjected to lymphography and dissection in order to identify lymphatics in the hindlimb. Six male mice aged 12 weeks were subjected to lymphadenectomy. The detail protocol of surgery was shown below.

### Dissection of Mice

The injection dye was composed of a mixed solution of 3%w/v Evans blue dye (Sigma-Aldrich Japan, Tokyo, Japan) and indocyanine green (ICG; Daiichi Sankyo, Tokyo, Japan) at a concentration of 2.5 mg/ml. The ratio of Evans blue dye to ICG was two to one. Mice were anesthetized with 2% isoflurane, and 15–30 μl of the injection dye was intradermally or subcutaneously administered at different areas (the footpad, ankle, back, or lower abdomen) to visualize lymphatics and LNs. Fifteen to 30 minutes after the injection under awakening, mice were euthanized via an intraperitoneal (IP) injection of a large dose of pentobarbital sodium (0.5 mg/g weight), and dissected to locate the lymphatics and LNs of interest.

### Lymphography by NIRF Imaging

Lymphatics were visualized using the NIRF imaging system (Pde-neo; Hamamatsu Photonics K.K., Shizuoka, Japan) after anesthetization with 1.5% isoflurane. Pde-neo emitted light at 760 nm and detected light at wavelengths greater than 820 nm, and, thus, detected ICG flow because ICG has an excitation wavelength of 805 nm and emission wavelength of 840 nm^18,32^. After an injection of 2 to 10 μl ICG, we massaged the injection site using absorbent cotton wool to promote ICG absorbance. Fluorescent images of lymph flow were obtained by Pde–neo.

### Surgical Procedures for Lymphadenectom**y**

Mice were anesthetized with 2% isoflurane. Iliac LNs were identified after the intradermally injection of 15 μl of 3%w/v Evans blue dye into the food pad. After 10 minutes, flunixin (Banamine®, 5 mg/kg weight; AdooQ BioScience, CA) and enrofloxacin (8.5 mg/kg weight; Wako Pure Chemical Industries, Ltd., Osaka, Japan) were injected subcutaneously as analgesics and antibiotics. A 1.0-cm incision into the abdominal muscle was made, the small intestine was pulled out, and the right iliac LN was excised using scissors. The small intestine was kept wet by applying saline at a temperature of 37 °C during surgery. After the excision of the iliac LN, 1 ml of sterile saline (0.9%) was injected intraperitoneally to support fluid homeostasis. Surgical wounds were secured using 6–0 nylon simple interrupted sutures, and surgical sites were then bonded together and dressed with gauze. Postoperative care was performed for these mice based on a previous study^33^. Flunixin (5 mg/kg weight) was administered subcutaneously twice per day (every 12 hours) for 7 days as analgesics, and 300 μl glucose (5%) and 300 μl saline (0.9%) were injected subcutaneously twice per day for 7 days as fluid therapy. Mice had free access to glucose (15%) offered in a second drinking bottle. One week after surgery, the inguinal and popliteal LNs were identified by a injection of 15 μl of 3% Evans blue dye solution into the ankle and food pad, delivered using a micro syringe with a 27 G needle. The surgical procedures for excising the inguinal LN were described in our previous study^32^. The right hindlimb was the operated side, while the left hindlimb was the control side. The control side did not undergo a sham operation (untreated). In our preliminary examination, sham operation using two mice did not occur edematous changes and these operation areas did not affect for histological evaluation (data not shown). Therefore, skin characteristics after the operation were compared with normal skin characteristics.

### Lymphedema Assessment

The circumferences of both hindlimbs were measured using adhesive tape (Transpore^TM^ surgical tape, Tokyo, Japan) to assess the extent of postsurgical edema. The degree of lymphedema was also quantified by measuring hindlimb volumes using a water displacement volumetry method as described previously^27^. The ratio of luminance (%) = total pixels of fluorescent areas on the lateral, front, and back sides in the hindlimb/total pixels of hindlimb areas on the lateral, front, and back sides. The ratio of luminance on day 60 was calculated to quantify the degree of lymphatic stasis after an injection of 2 μl ICG at the ankle on both hindlimbs on day 58. We did not massage the injection site in this evaluation.

Specimens of the hindlimb were stained immunohistochemically. The procedure used was described in detail below. The area of capillary lymphatics in the dermis (µm^2^/mm^2^) = the total area of capillary lymphatics in the dermis/mm^2^ dermis. The number of CD4+ cells (number/mm^2^) = the number of CD4+ cells/mm^2^ dermis and subcutaneous area. The ratio of collagen I (%) = pixels of collagen fibers/pixels of the dermis or subcutaneous area. These parameters were calculated by Adobe Photoshop Element 11.0 (Adobe System, Inc., Tokyo, Japan). The operated side was compared with the control side for all parameters.

### Immunohistochemistr**y**

After dissection, specimens of skin on the lower external hindlimb were harvested, stapled onto transparent plastic sheets to prevent excessive contractions, and fixed in 4% paraformaldehyde in 0.1 mol/L phosphate buffer (pH 7.4) overnight. Specimens were then placed in 10% sucrose in PBS for 2 hours, 20% sucrose in PBS overnight, and 30% sucrose in PBS overnight before embedding OCT compound (Sakura® Finetek, Tokyo, Japan). Frozen tissue was stored at −80 °C until used. Ten-micrometer-thick sections were mounted on MAS-coated glass slides (Matsunami Glass Ind., Ltd., Osaka, Japan) for hematoxylin and eosin (H&E) or immunostaining. Sections for immunostaining were washed with PBS/0.03% Tween-20 (PBST), incubated with blocking solution (X0909, Dako, CA) and 0.3% hydrogen peroxide, and then incubated with primary antibodies diluted in PBST at room temperature (RT) for 2 hours. Sections were washed three times using PBST. They were then incubated with the secondary antibodies linked with HRP at RT for 1 hour. The primary antibodies used in the present study were an anti-LYVE-1 antibody (ab14917, Abcam), anti-CD-4 antibody (ab25475, Abcam), and anti-collagen I antibody (ab34710, Abcam).

### Statistical Analyses

Data were compared using the paired *t*-test with JMP 8.0.1 (SAS, USA). Results were expressed as the mean ± SD. A P-value of less than 0.05 was used as the criterion to indicate significance.

## Electronic supplementary material

Video 1

Video 2

Supplementary information
