# Supplementary material for: Near-Infrared Fluorescence Imaging Directly Visualizes Lymphatic Drainage Pathways and Connections between Superficial and Deep Lymphatic Systems in the Mouse Hindlimb
Source: Sci Rep. 2018 May 4;8:7078. doi: 10.1038/s41598-018-25383-y (PMC5935665; doi:10.1038/s41598-018-25383-y)
Supplement: Supplementary file 3 — Supplementary information [file 41598_2018_25383_MOESM3_ESM.doc]

**Supplementary Information**

**Near-Infrared Fluorescence Imaging Directly Visualizes Lymphatic Drainage Pathways and Connections between Superficial and Deep Lymphatic Systems in the Mouse Hindlimb**

*Yukari Nakajima, Kimi Asano, Kanae Mukai, Tamae Urai, Mayumi Okuwa, Junko Sugama, Toshio Nakatani*

**Supplemental video 1. The deep medial system in the mouse hindlimb.**

The deep medial system drains lymph fluid from the hindlimb into the abdominal cavity (white closed arrowhead). Fluorescence flow was easily detected along with the femoral artery.

**Supplemental video 2. Connection between superficial and deep lymphatic systems in mice.**

Lymphatics between the inguinal and iliac LNs were detected by stretching the skin to the sagittal line (closed red arrowhead). Fluorescence flow drained from the superficial (subcutaneous) to the deep (abdominal cavity) lymphatic system.
